# Clinical and imaging analysis of subclinical hemophilia combined with coxarthrosis: case report and literature review

**DOI:** 10.1186/s40064-016-3727-7

**Published:** 2016-12-01

**Authors:** Cheng Liu, Jun Guo, Qiu Cui, Dingfeng Li, Yanjun Zeng

**Affiliations:** 1 Department of Orthopedics, The Affiliated Hospital of Academy of Military Medical Sciences, PLA 307th Hospital, Beijing, 100071 China; 2 Beijing University of Technology, 100 Pingleyuan, Chaoyang District, Beijing, 100022 China

**Keywords:** Subclinical hemophilia, Coxarthrosis, Arthritis

## Abstract

**Introduction:**

The incidence of subclinical hemophilic arthritis is low, with this case reports and literature review, we hope clinicians could pay more attention to the diagnosis of subclinical hemophilic arthritis and prevent the misdiagnosis and mistreatment.

**Case presentation:**

We analyzed the imaging feature, and therapy of a subclinical hemophilia case with hip lesion by reporting its clinical manifestations, imaging features and therapy procedure, and reviewing literatures.

**Results:**

Hemophilia is a sex-linked recessive hereditary hemorrhagic disease, and the pathogenesis of subclinic hemophilia is concealed, which often involves joint lesion. The imaging of joint lesion of hemophilia cases is typical, which facilitates the differential diagnosis with other joint diseases. The current treatment is mainly supplementary or replacement therapy.

**Conclusion:**

Hemophilic arthritis cases, especially the mild or subclinical cases without family history and with an older age of onset, are not common. The disease should be further understood, and early diagnosis and treatment is crucial to prevent the progressive development of joint diseases.

## Background

Hemophilia is a sex-linked recessive hereditary hemorrhagic disease that can be divided into three types, namely hemophilia A, B and C. The most frequent is hemophilia A caused by factor VIII deficiency. According to factor VIII levels, the hemophilia cases are divided into severe (<1%), moderate (1–5%), mild (5–25%) and subclinical (25–45%). The incidence of hemophilia in social groups is 5–10 per 100,000 persons and in infants is about 1/5000, without significant regional and ethnic difference (Zimmerman and Valentino 2013). Joint bleeding is one of the typical symptoms of bleeding in pediatric hemophilia, with an incidence of 70–80% (Deng et al. 2001). The cases with coagulation factor VIII levels higher than 20% usually do not develop into hemophilic arthritis regardless of the history of joint bleeding (Stein and Duthie 1981). However, nearly one-third of cases with coagulation factor VIII levels between 6 and 20% suffer from chronic arthritis (Stein and Duthie 1981). We searched Pubmed for articles with abstract and keywords using “mild”, “hemophilic arthritis” as the search words, so far only two reports were found. Therefore, mild and subclinical hemophilic arthritis are rarely reported. In the present article, one case of subclinical hemophilic coxarthritis found in our hospital is reported, and a literature review is also performed.

## Case description

A male patient, aged 13, was hospitalized in February mainly due to aggravated kinesalgia of left knee for 1 year and 9 months, which was accompanied by left lower limb lameness for two months. The pain was present when the patient was active and was relieved after rest. Fever, night sweats, nausea, vomiting, night pain and other symptoms were not concurrent. The symptoms were worsened due to strenuous exercise 2 months ago. X-ray and MRI examinations of left knee performed in other hospital revealed no abnormalities; CT and MRI examinations of bilateral hip joints revealed abnormalities. CT images showed that bilateral acetabular articular surfaces were rough with increased densities; cortical bone fractures and small free bone fragments were observed; the left femoral head was irregular with rough edges and unclear bone trabeculae; the right femoral head was basically normal. MRI examinations of bilateral hip joints showed that bilateral hip joints were in asymmetry; a small amount of hydrops were present in the left hip joint cavity; the joint space was widened, the acetabulum became shallow; the femoral head was slightly shifted upward, the femoral head and acetabular articular surface were rough and irregular; areas with abnormal signal intensity were observed in local sclerotins; T1W1 scan showed uneven and slightly lower signal intensities; T2W1 and STIR scans showed heterogeneous signal hyperintensity; the peripheral soft tissues were swollen with irregular heterogeneous signal intensity. The right hip joint space was present, the superior margin of the acetabular articular surface was rough; abnormal intensities were not observed in the right femoral head or in the upper segment of the femur and acetabulum. The imaging findings were as follows: (1) Right hip joint dysplasia; (2) Left hip joint abnormalities. Infectious disease, preferably the joint tuberculosis was considered. Aseptic necrosis of the femoral head was not excluded. The physical examinations on admission showed that the left lower limb lameness was present, tenderness of bilateral hips and inguinal region was absent, left patrick sign was positive, external rotation of left hip was obviously limited. The circumferences of the left and right knees at 15 cm above the patellas were 38 and 41 cm respectively. The distance from the anterior superior iliac spine to the medial malleolus was 82 cm for the left side, and 83 cm for the right side. Bilateral lower limbs had muscle strengths of Class V, symmetrical sensations, normal physiological reflections and negative pathological signs (−).

**Fig. 1:**
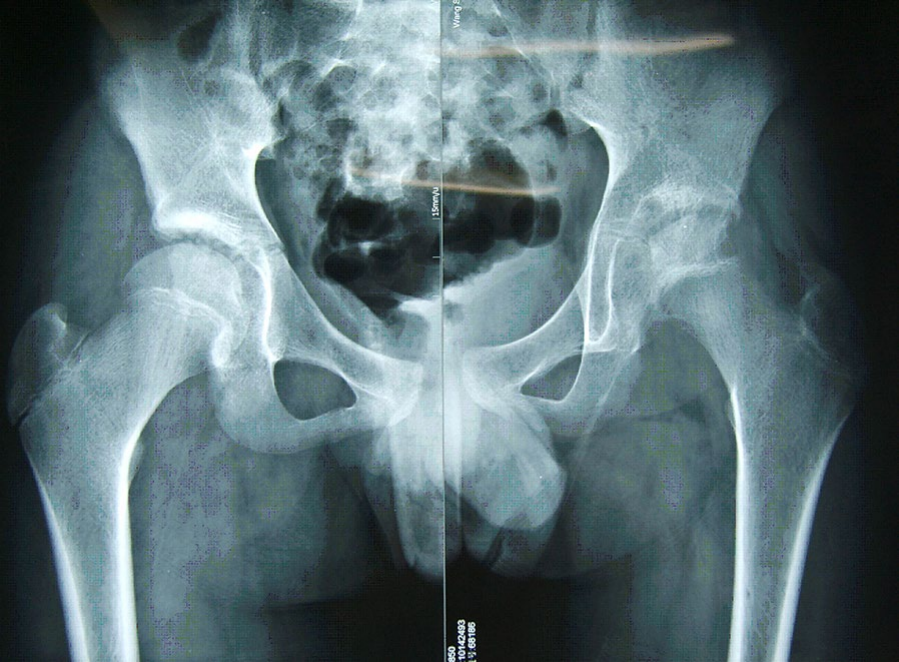
X-ray plain film of bilateral hip joints. The left femoral epiphysis was irregular with bone defect areas at both outer and inner margins; the space of the left hip joint had local stricture; peripheral spindle-shaped soft tissue density shadows were observed. The right acetabulum was thickened and the density was increased

**Fig. 2:**
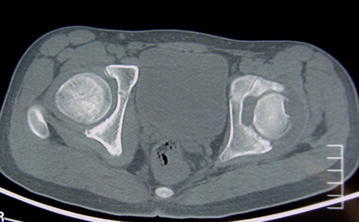
CT images of bilateral hips. The left femoral head was irregular with rough margins, the bone trabeculae were unclear; cortical bone fracture and small free bone fragments were observed

**Fig. 3:**
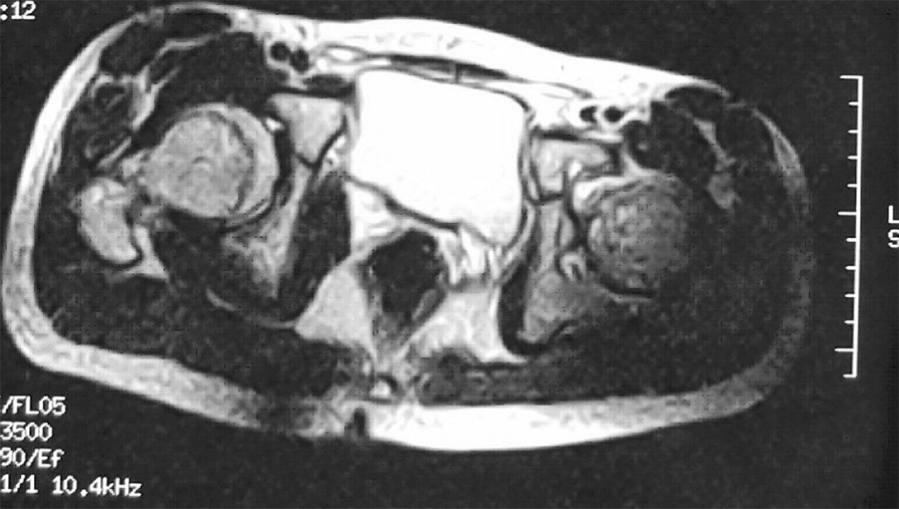
The horizontal MRI of bilateral hip joints, the femoral head and acetabular articular surfaces were rough

**Fig. 4:**
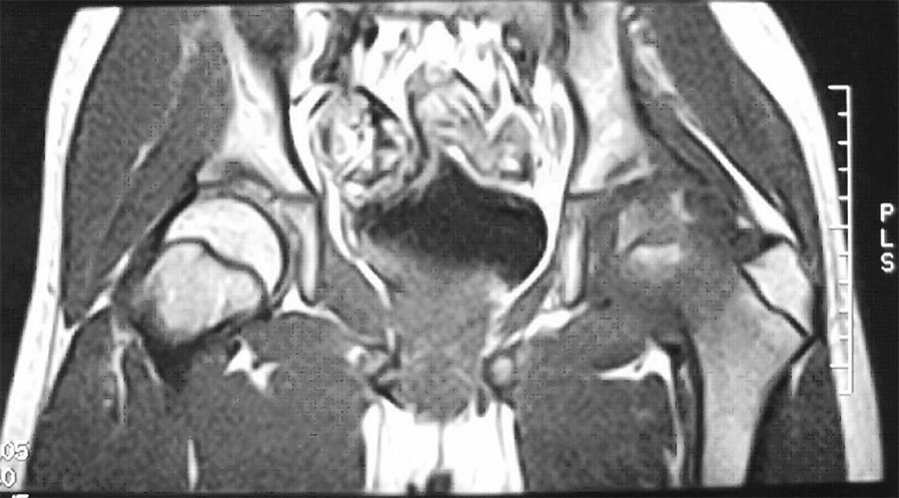
The coronal MRI of bilateral hip joints, the left acetabulum became shallow, the femoral head was shifted upward slightly

After admission, the pelvic X-ray examinations showed that the left femoral epiphysis was irregular with bone defect areas on both outer and inner margins; the local bone density was increased, local bone defects were also observed on the outer margin of the femoral neck without obvious periosteal reactions. The left acetabulum was inhomogeneous in density and had multiple saccular low-density shadows. The space of the left hip joint was uneven with local stricture; peripheral spindle-shaped soft tissue density shadows were observed. The right acetabulum was thickened and whitened, and multiple mottled shadows with low signal densities were observed. The right hip joint space was not narrow. Other parts of bilateral hip joints had no obvious bone abnormalities. The findings also indicated high possibility of hip joint tuberculosis. The imaging data are shown in Figs. 1, 2, 3, 4.

After admission, routine examinations were conducted. Electrocardiogram and abdominal B-type ultrasonography showed no abnormalities; the test results of hemogram, liver function, renal function and erythrocyte sedimentation rate were normal. Blood biochemistry tests showed that the serum C-reactive protein level was 7.08 mg/L (Reference value: 0–3 mg/L). In the four indices of coagulation function, the partial thromboplastin time was 38.7 s (Reference value: 24.9–36.8), and the other three indices were normal. The tuberculin test showed a strongly positive result. After admission, the patient underwent diagnostic puncture of the left hip. The result indicated the presence of uncoagulated bloody liquid. The coagulation factors were further detected in Institute of Hematology, the Peking Union Medical College Hospital. The results indicated that the value of factor VIII was 27.8 (Reference value: 50–100), both factors IX and XI were normal. Therefore, the patient was suspected as subclinical hemophilia. After the patient was infused with cryoprecipitate blood preparation, reexamination of the blood gave a normal result, and the local pain was quickly relieved.

## Summary and analysis

### Pathogenesis

The vast majority of patients with hemophilia are of hemophilia A type, which is a sex-linked recessive hereditary hemorrhagic disease due to deficiency of coagulation factor VIII, i.e., antihemophilic factor (AHF). Factor VIII is a complex composed of rocoagulant components factor VIII (VIII: C) and factor VIII-related antigen (VIII R: Ag). F VIII gene is quite large. It is located on Xq28 and consists of 26 exons and 25 introns with a full length of 186 kb. Nearly 300 types of mutations of F VIII gene have been found. The genotype is closely related to the phenotype. Point mutations and gene deletions cause mild F VIII deficiency. Deletion of termination codon and major insertions, shifts and nonsense mutations usually cause severe FVIII deficiency and clinical phenotype (Mannucci et al. 2012).

### Clinical manifestations and diagnosis

The patients often suffer from bleeding due to microtrauma or frequent epistaxis. The poor blood coagulation after bleeding is usually the major sign. The uncoagulated hemorrhage leads to the rapid increase of intra-articular pressure, thereby causing abrupt joint swelling, pain and limited mobility. Diagnostic criteria of hemophilia includes: (1) Clinical manifestations of hemophilic bleeding; (2) Abnormal results of certain laboratory tests. For example, the procoagulant activity of F VIII (F VIII: C) is low, or the APTT is prolonged. However, the prothrombin time, total clotting time, bleeding time and latelet count were all normal in this patient. (3) Typical family history of hemophilia. Some patients do not have a typical family history. These cases may be associated with the F VIII gene mutations.

### Hemophilic arthritis

The most frequently affected joint is knee joint, followed by the elbow joint, ankle joint, hip joint and shoulder joint. A single lesion is usually involved in early stage of the disease. Later, other joints are gradually affected. Repeated joint bleeding can cause chronic injury, synovitis, articular cartilage damage, hyperostosis, atrophy of the joint, lip-like hyperplasia and osteophyte formation on the joint surface, stricture of joint cavity, bone necrosis and cystic degeneration, which eventually lead to joint deformity and dysfunction. It is commonly believed that the patients with severe hemophilia are prone to spontaneous joint bleeding because the factor levels are very low. The mild and moderate patients with slightly higher factor levels have a lower rate of spontaneous bleeding. But it is recently reported that the degree of joint damage in mild or subclinical hemophilia cases cannot be evaluated only through cytokine levels due to the lack of imaging data (Di Minno et al. 2013).

#### Pathological changes of hemophilic arthritis

Basic pathological features of hemophilic arthropathy include spontaneous intra-articular bleeding or microtrauma-induced bleeding. A small amount of bleeding for the first time can be completely absorbed without leaving a trace, but long-term, repeated and large amounts of bleeding can result in a series of pathological changes of synovium, articular cartilage and subchondral bone, and even cause joint stiffness and deformity. Stimulation of iron deposition will lead to synovitis accompanied by release of cytokines. Repeated synovitis will lead to progressive damage of cartilage and subchondral bone.The joint space will be narrowed with the progression of the disease. The joint damage, angulation and joint ankylosis may occur at the final stage. So far, bone and cartilage destruction is believed to be irreversible lesion. However, other changes such as oozing, bleeding and ynovial thickening are recoverable (Brummel-Ziedins et al. 2009).

#### X-ray findings of hemophilic arthritis

X-ray findings of hemophilic arthropathy depend on the bleeding site and different pathological stage. The bone and joint changes in hemophilia are directly or indirectly related to the intra-articular, circum-articular or intraosseous bleeding. Early bleeding is confined to soft tissues. Therefore, only joint swelling and increase of concentration can be observed. When chronic arthritis occurs, the cartilage and bone destruction is concurrent. X-ray image usually indicate the signs such as stricture of joint cavity due to degradation and absorption of articular cartilage. Generally, the hemophilic arthritis in diagnostic X-ray is characterized by enlarged and squared epiphysis, defective or wavy epiphyseal line, and swelling of joint capsule with increased density. Joint space narrowing, epiphysis destruction, formation of saccular absorption area and osteoporosis are commonly observed. The Pettersson scoring system based on the severity of X-ray changes has been applied since early 1980s. This scoring system is used to sequentially record the imaging abnormalities based on the method of clinical classification combined with radiological classification. The sum of various scores represents the total scores of each examination. This scoring method has been currently adopted by the World Federation of Hemophilia, because of the detailed and comprehensive evaluations on joint disease. However, the X-ray examination has its limitations in that it cannot show the early abnormal signs of joint, differentiate between the articular dropsy and bleeding in soft tissue swelling or directly display the pathological changes of synovium and cartilage. Therefore, Pettersson scoring system cannot be used to evaluate the pathological changes in the soft tissues, joint effusion and synovitis. Additionally, X-ray imaging cannot directly show the articular cartilage and the changes can be only presumed through joint space narrowing. Therefore, X-ray examination and scoring is more applicable for the hemophilic arthropathy with bone lesion (Christoforidis et al. 2011).

#### MRI changes in hemophilia arthritis

MRI examination is advantageous in displaying hydrarthrosis/hemarthrosis, synovial thickening/hemosiderin deposition, erosion of the joint edge/subchondral cyst. It can also display some articular cartilage loss/joint degeneration. It is especially beneficial for displaying chronic synovitis and synovial thickening in patients of emophilia. MRI examination can provide a higher resolution, reveal early joint lesions, and discover more progressive lesions of bone and cartilage than plain film X-ray. Of several MRI scoring methods, the most commonly used is the Denver scoring (Nuss et al. 2000), the European scoring (Lundin et al. 2004) and MRI scoring based on progression compatible with additional signs (Lundin et al. 2005). The MRI scoring system established by the World Federation of Hemophilia (WFH) and International Prophylaxis Study Group (IPSG) is the newest scoring system (Lundin et al. 2012). This system comprehensively assesses and quantifies the severity of joint disease based on the lesions of soft tissues and osteochondrosis. It can discover the small lesions in the articular surface by specifying the observation indicators of lesions in articular soft tissues and articular cartilage and make the efficacy evaluation more convenient by quantifying the data before and after treatment.

## Discussion

### Differential diagnosis

The early manifestation is similar to that of serous or purulent synovitis, so it is difficult to be distinguished from the articular hematoma after the epiphyseal injury. Cystic degeneration of the bones in the aggressive phase of hemophilic osteoarthritis is more common than that in osteoarthritis. It is difficult to discriminate hemophilic osteoarthritis from rheumatoid arthritis when osteoporosis is combined. Typically, a definite diagnosis can be made by reference to clinical history and laboratory tests, also the differential diagnosis can be made by the presence of hemosiderin in MRI.

### Treatment

Topical hemostasis and systemic replacement therapy are available. For the patients with obvious joint swelling and pain, joint puncture and aspiration technique should be performed under the conditions of factor VIII supplement and strict local disinfection to reduce stress, relieve pain and reduce the damage of hematocele to articular cartilage and bone. To prevent local infection, chronic fistula formation and fatal septicemia, aspiration, drainage and biopsy should not be performed during treatment. For the late phase joint disease, synovectomy, joint irrigation, arthrodesis or arthroplasty should be performed depending on the severity of the lesions (Auerswald et al. 2012). Replacement therapy is still preferred in systemic treatment. The factor replacement therapy can be divided into preventive treatment and the treatment based on specific needs of the patients. In 1994, WFH (International Federation of Hemophilia) and WHO established the early preventive treatment as the best treatment protocol for pediatric patients with severe hemophilia. Specifically speaking, the systemic therapy includes the following aspects: (1) Replacement therapy: fresh frozen plasma; cryoprecipitate; genetic engineering products of F VIII; F VIII preparations from animal; recombinant activated factor VIIα preparations; prothrombin complex concentrate; FIX preparations with high purity. (2) Medication-assisted therapy: ranitidine, desmopressin, danazol, antifibrinolytic agents and carbazochrome salicylate. (3) Spleen cell transplantation or spleen transplantation. (4) Gene therapy. Since 1970s, the morbidity and mortality of hemophilia has been greatly reduced with the effective application of replacement treatment. Since 1980s, the safety of replacement treatment is guaranteed with the continuous improvement of virus inactivation technology, thereby greatly reducing the incidence of viral hepatitis and AIDS in hemophilia. Since the 1990s, the widespread application of recombinant coagulation factors not only improves the efficacy, but also avoids the side effects of antibody production and viral infection caused by the application of traditional human-derived clotting factors. Gene therapy achieves success in animal trials. The results of gene therapy in preliminary clinical trials also bring hope to hemophilia patients (High 2014).

Generally speaking, the patients with severe hemophilia are prone to spontaneous joint bleeding due to very low factor levels. The patients with mild and moderate hemophilia have lower incidence of spontaneous bleeding due to slightly higher factor levels. It is reported that subclinical cases usually suffer from bleeding after severe trauma or surgery, but joint diseases of those cases are rarely reported. Therefore, we speculate that the present patient suffered from the disease due to repeated strenuous exercise. Based on our analysis, the diagnosis of the patient was delayed due to the following reasons: (1) The patient suffered from hip disease but complained of knee pain. Inspections of the knee without careful physical examinations could not find the real lesion; (2) Collection of medical history data was incomplete. Most patients with hemophilia had history of spontaneous bleeding or continuous bleeding after minor injury. Frequent gingival hemorrhage when brushing was found when the patient was questioned insistently for the history; but the patient had no family history. The clinical data have shown that about 70% patients with hemophilia have a family history, which is not confirmed in about 30% patients; (3) The patient was relatively senior. Hemophilia patients showed first signs at the age of 3. The patient had a relatively older age of onset because he was a subclinical case, and the joint bleeding is caused by strenuous exercise; (4) Examinations after admission showed that the clotting time was basically normal; (5) The physical examination revealed that the patient had a strongly positive tuberculin test without abnormalities in chest radiography or history of low fever and night sweats, and the erythrocyte sedimentation rate was normal. Therefore, the possibility of tuberculosis was low.

In summary, the presence of hemosiderin within the joint seen in the MRI, but radiological changes presuming the final diagnosis of hemophilia. Furthermore, the joint disease caused by subclinical hemophilia has been rarely reported. Bloody fluids were aspirated from the patient by joint puncture, and then correct diagnosis was obtained through specific examinations. Therefore, more attention should be paid to the combination of clinical and imaging examinations in the future so as to achieve early diagnosis and timely treatment of hemophilic arthritis, especially the mild and subclinical hemophilic arthritis. This is important to avoid progressive joint destructions or further bleeding due to unnecessary surgery.
